# Supplementary material for: SVvalidation: A long-read-based validation method for genomic structural variation
Source: PLoS One. 2024 Jan 5;19(1):e0291741. doi: 10.1371/journal.pone.0291741 (PMC10769053; doi:10.1371/journal.pone.0291741)
Supplement: S1 File — (PDF) [file pone.0291741.s001.pdf]

SVvalidation: a long-read based validation method for genomic structural variation-supplementary file

Yan Zheng and Xuequn Shang

September 12, 2023

1 Data sources and program commands

1.1 Data sources

The long-read download links and reference download links are shown in Table S1. The repeat region of GRCh37 and GRCh38 was downloaded from http://genome.ucsc.edu/cgi-bin/hgTables. We choose parameter simple repeat and output in bed file format.

1.2 Commands for generating the bam files

The below commands are used to generate bam files from fastq files of CHM13 and HG002.

minimap2 -ax map-ont –MD GRCh37.fa hg002_ont.fastq.gz >hg002_ont.sam
samtools view -bS hg002_ont.sam >hg002_ont.bam

minimap2 -ax map-pb –MD GRCh37.fa hg002_pacbio.fastq.gz >hg002_pacbio.sam
samtools view -bS hg002_pacbio.sam >hg002_pacbip.bam

minimap2 -ax map-ont –MD GRCh38.fa chm13_ont.fastq.gz >chm13_ont.sam
samtools view -bS chm13_ont.sam >chm13_ont.bam

minimap2 -ax map-pb –MD GRCh38.fa chm13_pacbio.fastq.gz >chm13_pacbio.sam
samtools view -bS chm13_pacbio.sam >chm13_pacbio.bam

1.3 Commands for Assemblytics

The below commands are used to generate the list of SVs from the reference genomes.

./nucmer -maxmatch -l 100 -c 500 GRCh38.fa chm13.fa -prefix chm13_OUT

./Assemblytics chm13_OUT.delta $\langle chm13\_output \rangle$

1.4 Commands for SVvalidation and vapor

We run SVvalidation and vapor using the following commands.

./SVvalidation input.bam input.bed

./vapor bed –sv-input ../input.bed –output-path ../vapor_result/ –reference ../reference.fa –pacbio-input ../sample.bam

1.5 Commands for cuteSV and Sniffles

We run cuteSV, Sniffles2 and DeBreak using the following commands.

Table S1: The download link of different datasets

datasets	coverage	link
HG002 ONT	50x	https://nist-midas.s3.amazonaws.com/pdrsrv/mds2-2336/input_fastqs/HG002_GM24385_1_2_3_Guppy_3.6.0_prom.fastq.gz
CHM13 ONT	107x	https://s3-us-west-2.amazonaws.com/human-pangenomics/T2T/CHM13/nanopore/rel3/rel3.fastq.gz
HG002 pacbio	37x	https://nist-midas.s3.amazonaws.com/pdrsrv/mds2-2336/input_fastqs/HG002_35x_PacBio_14kb-15kb.fastq.gz
CHM13 pacbio	34x	https://s3-us-west-2.amazonaws.com/human-pangenomics/T2T/CHM13/pacbio/hifi_20kb/m64062_190806_063919.subreads.bam
GRCh37 ref	null	http://hgdownload.cse.ucsc.edu/goldenPath/hg19/bigZips/hg19.fa.gz
GRCh38 ref	null	https://hgdownload.soe.ucsc.edu/goldenPath/hg38/bigZips/hg38.fa.gz

./cuteSV ⟨*input_bam*⟩ ⟨*reference*⟩ ⟨*output_file*⟩ ⟨*work_dir*⟩
./sniffles2 -m ⟨*input_bam*⟩ -v ⟨*output_file*⟩
./debreak –bam ⟨*input_bam*⟩ -o ⟨*output_dir*⟩

2 The detailed data for validation result

In the main manuscript, we show the F1 score of SV-validation and vapor in HG002 and CHM13. In the HG002 benchmark, there are 5463 DELs and 7279 INSs. In the CHM13 benchmark, there are 3647 DELs and 6457 INSs. We use these SVs to check the recall value of methods. At the same time, we simulated some false SVs and use these false SVs to check the precision value. Finally, we calculate F1-score by the recall and precision value. The detailed number are shown in the following Table S2, Table S3,Table S4,.

3 The detailed step to get the SVs in repeat regions

In the main manuscript, we observed that the vapor has a good performance in normal regions, but nor perform well in repeat regions. The repeat region of GRCh37 and GRCh38 are downloaded from table browser (The website is http://genome.ucsc.edu/cgi-bin/hgTables). On the website, we download the GRCh37 repeat regions by setting parameters just like the Figure S1.

From the previous step, we get the list of repeat regions (GRCh37 and GRCh38). Then we compare the SVs with the repeat regions. For a SV_i (chr_i,pos1_i,pos2_i, SV_len_i), if we can find a repeat_region_j that satisfies the following criteria, we consider the SV_i to be a SV in the repeat region.

For the repeat_region_j (chr_j,start_j,end_j):

1. chr_i is same with chr_j.
2. min(pos2_i,end_j)-max(pos1_i,start_j) > 0.8*SV_len_i

These two criteria can be understood as, if over 80% part of a SV is in a repeat region, we consider that the SV is in the repeat regions.

4 Methods for checking whether SV caller output a SV in the benchmark

For different SV callers or benchmarks, they may report the same SV with slightly different positions and slightly different lengths. It is not easy to determine if two SVs are the same or not. Below, we describe a simple method to judge whether two SVs are the same SV.

4.1 Comparing two DELs

Consider two deletions DEL1: (start1_pos, end1_pos) and DEL2: (start2_pos, end2_pos). Set DEL1_len = end1_pos - start1_pos + 1 and DEL2_len = end2_pos - start2_pos + 1.

DEL1 and DEL2 are said to be the same if:

Table S2: The recall of different methods

methods	HG002_ONT_del	CHM13_ONT_del	HG002_pacbio_del	CHM13_pacibo_del	HG002_ONT_ins	CHM13_ONT_ins	HG002_pacbio_ins	CHM13_pacbio_ins
SVvalidation	98.0%	97.2%	96.6%	94.9%	98.9%	97.7%	95.8%	94.3%
vapor	83.9%	83.3%	73.8%	75.4%	78.7%	86.9%	79.3%	83.4%

Table S3: The precision of different methods

methods	HG002_ONT_del	CHM13_ONT_del	HG002_pacbio_del	CHM13_pacibo_del	HG002_ONT_ins	CHM13_ONT_ins	HG002_pacbio_ins	CHM13_pacbio_ins
SVvalidation	87.1%	88.9%	90.8%	88.2%	97.6%	98.4%	94.8%	90.4%
vapor	69.5%	73.3%	74.4%	76.3%	94.8%	95.3%	92.3%	88.6%

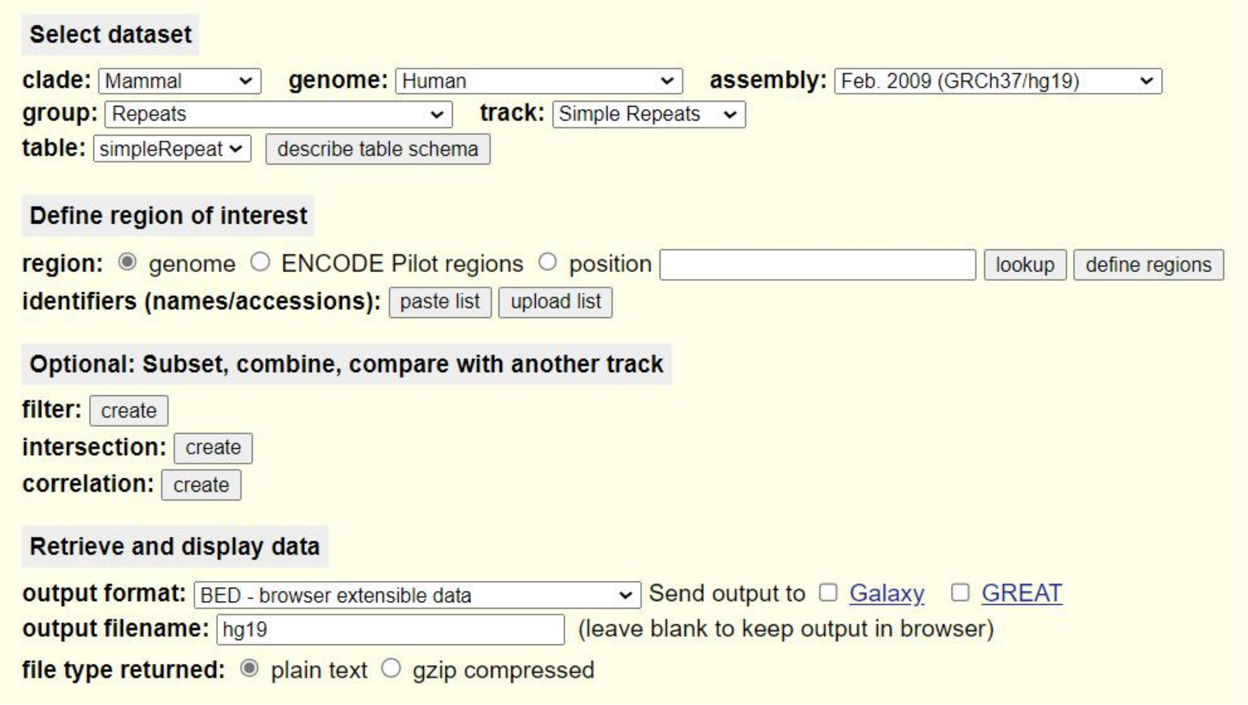

Figure S1: The simple repeats parameters are shown in figure.

1. max(start_pos1,start_pos2)-min(end_pos1,end_pos2) less than 1000.
2. abs(DEL1_len-DEL2_len) <0.5*min(DEL1_len,DEL2_len).

4.2 Comparing two INSs

Consider two insertions INS1: (bp1, INS1_len) and INS2: (bp2, INS2_len)

INS1 and INS2 are said to be the same if:

1. abs(bp2-bp1) less than 1000.
2. abs(INS1_len-INS2_len) <0.5*min(INS1_len,INS2_len).

5 Methods for creating false SVs list

In order to test the performance of the validation method, we create a list of false SVs that is harder

Table S4: The F1-score of different methods

methods	HG002_ONT_del	CHM13_ONT_del	HG002_pacbio_del	CHM13_pacibo_del	HG002_ONT_ins	CHM13_ONT_ins	HG002_pacbio_ins	CHM13_pacbio_ins
SVvalidation	0.92	0.93	0.94	0.91	0.98	0.98	0.95	0.92
vapor	0.76	0.78	0.74	0.76	0.86	0.91	0.85	0.86

to filter. For each sample, we Randomly select 2000 true DELs and 2000 true INSs from the benchmark. Next, we create 4000 false SVs for the 4000 true SVs. The detailed method to create false DELs and INSs is shown in the following steps:

For DEL_i: (chr_i,pos1_i,pos2_i,SV_len_i)

1. We randomly select a number from [0.1,0.2,0.3,0.4,2,3,4,5], labeled as num_i.
2. Next, we calculate a false SV length by SV_len_i*num_i, labeled as false_len.
3. Next, we record the false DEL by a turple (chr_i,pos1_i,pos1_i+false_len,false_len)

For INS_i: (chr_i,pos1_i,SV_len_i)

1. We randomly select a number from [0.1,0.2,0.3,0.4,2,3,4,5], labeled as num_i.
2. Next, we calculate a false SV length by SV_len_i*num_i, labeled as false_len.
3. Next, we record the false INS by a turple (chr_i,pos1_i,false_len)

For these false DELs and false INSs, they have at least 50% length error. If validation methods still validate them as correct SV, we think that the method outputs false positive SVs.
